# Lipidomic and Proteomic Profiling of the Milk Fat Globule Membrane from Different Industrial By-Products of the Butter and Butter Oil Manufacturing Process

**DOI:** 10.3390/foods12040750

**Published:** 2023-02-08

**Authors:** María Señoráns, Veronica Gallo, María V. Calvo, Javier Fontecha

**Affiliations:** Food Lipid Biomarkers and Health Group, Institute of Food Science Research (CIAL, CSIC), Nicolás Cabrera 9, 28049 Madrid, Spain

**Keywords:** milk fat globule membrane (MFGM), polar lipids, buttermilk, butterserum, MFGM proteins

## Abstract

Recent studies have demonstrated the positive effects of regular intake of milk fat globule membranes (MFGMs) on neural and cognitive development, as well as immune and gastrointestinal health in infants and elders. Dairy products and by-products generated from the butter and butter oil manufacturing process are valuable sources of MFGM. Thus, in view of the growing need to reduce by-products and waste, it is crucial to foster research aimed at the valorization of dairy by-products rich in MFGM. For this purpose, all the by-products coming from butter and butter oil production (from raw milk to the related by-products) were used to study the MFGM isolated fractions, followed by their characterization through a combined lipidomic and proteomic approach. The patterns of polar lipids and proteins indicated that buttermilk (BM), butterserum (BS), and their mix (BM-BS blend) are the most suitable by-products to be employed as starting material for the isolation and purification of MFGMs, thus obtaining MFGM-enriched ingredients for the manufacture of products with high biological activity.

## 1. Introduction

Increasing research is being devoted to milk fat globule membrane (MFGM), that is, the protective three-layered membrane of milk fat globules (MFG) [1]. MFGM is 10–50 nm thick and contains a complex mixture of lipids (30–75%) and proteins (25–70%) [2]. Lipids represent the major building components of MFGM and include neutral lipids (NLs) and polar lipids (PLs). NLs comprise esters, cholesterol, mono-, di and triglycerides that make up the MFGM core, while PLs are, in particular, membrane phospholipids (phosphatidylethanolamine PE, phosphatidylcholine PC, phosphatidylserine PS, phosphatidylinositol, PI) and sphingolipids (mostly sphingomyelin, SM) [3,4]. Moreover, numerous proteins are associated with the MFGM, part of which are exposed on the inner face (e.g., xanthine dehydrogenase/oxidase, adipophilin), some at the outer part (e.g., Periodic acid/Schiff 6/7) while others span the entire membrane (e.g., butyrophilin, cluster of differentiation 36, mucin1, mucin15) [5]. Both lipids and proteins contribute to the nutritional, functional, and health benefits recognized in MFGM. In fact, recent studies demonstrated the positive effects of regular MFGM intake on neural and cognitive development as well as immune and gastrointestinal health. For instance, MFGM has been associated with decreased diarrhea and infections in infants and children and inhibition of tumor growth [6,7,8]. Furthermore, MFGM was reported to enhance milk stability, along with the whipping and water-holding capacity of dairy products [9,10,11]. For all these reasons, the prospects of using MFGM as an ingredient in food and non-food products is attracting notable interest [12]. Products and by-products generated by the dairy industry provide valuable sources of MFGM. Thus, faced with the increasing demand of dairy products and the simultaneous need to reduce the amount of by-products and waste, many research groups are now focusing on the valorization of dairy by-products rich in MFGM [13]. The major by-products of the dairy industry are cheese whey, skimmed milk, buttermilk (BM), and butterserum (BS). Specifically, cheese whey is the by-product of cheesemaking and skimmed milk is obtained from cream manufacture, while BM and BS consist of the aqueous phases released during butter and butteroil production, respectively [1,14]. In recent years, much attention has been paid to BM and BS as potential sources for the isolation of MFGM. Both contain higher amounts of MFGM proteins compared to whey or skimmed milk [2]. However, BS, which comes from anhydrous milk fat production, has higher fat content (up to 4.5%) than BM [15,16]. To date, various fractionation and concentration methods have been tried, each one with specific consequences on MFGM yield, structure, and/or composition. These features may in fact considerably change depending on the conditions applied during both raw material processing and the consequent MFGM isolation and purification procedures [17,18]. As an example, it has been widely demonstrated that heat treatments such as pasteurization (80 °C for 3 min) and ultra-heat treatments (125–145 °C) may significantly reduce the amount of some native MFGM protein, while cream churning may promote the destabilization of MFG and the consequent release of MFGM fragments into BM [19,20]. Overall, MFGM isolation consists in separating MFGM from other components such as whey proteins, caseins, lactose, and minerals. The main issues related to this process are the similarity in size of MFGM fragments to casein micelles as well as the association of whey proteins with MFGM during heating [21,22,23]. Since both can cause a reduction in the yield of MFGM proteins in the final isolate [2], MFGM separation and purification often require pre-treatment to remove casein (e.g., acid precipitation, enzymatic hydrolysis, filtration, washing with salt solution) and whey protein (e.g., filtration, washing with salt solution). One of the most frequently used methods to separate MFGM from whey consists of the addition of sodium citrate followed by microfiltration (MF) or ultrafiltration (UF). In fact, sodium citrate promotes the dissociation of casein micelles together with the permeation of a large amount of whey protein [24]. The wide variety of MFGM sources and conditions applied during MFGM recovery, such as type of pre-treatments, pore size of filtration membranes, centrifugation speed, and temperature, among others, not only affects the final yield of MFGM and its composition, especially lipid content and protein profile, but also makes it difficult to compare the results obtained in different pre-clinical and clinical studies. The composition of MFGM isolated from cheese whey and BM has already been extensively studied [16,25,26,27]. As indicated, although the main industrial source for obtaining MFGM is cheese whey, it is the by-products of butter and butter oil processing from which a higher yield can be obtained. However, it is not well known which among BM, BS, or their mixture is the most suitable for the isolation and purification of MFGM. For this purpose, it is crucial to investigate the lipid and protein MFGM fractions, both in qualitative and quantitative terms, of all by-products involved in butter and butter oil production (from raw milk to related by-products), followed by their characterization using a combined lipidomic and proteomic approach.

## 2. Materials and Methods

### 2.1. Materials and Reagent

**Figure 1:**
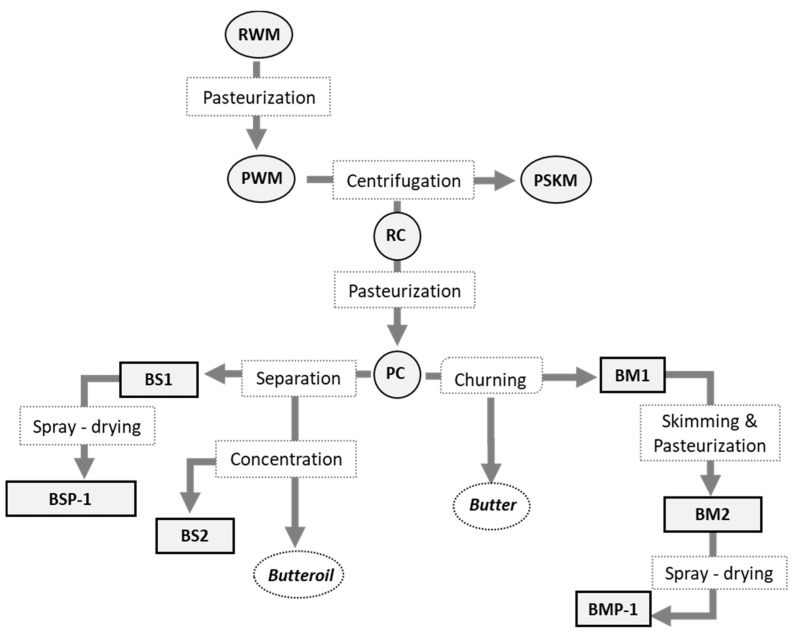
Flowchart of the studied samples obtained from butter and butter oil processing. At least two replicates of each type of sample were studied.

Milk, cream, and dairy by-products obtained from the industrial process of butter and butteroil manufacture (Figure 1) were provided by a dairy company (Industrias Lácteas Asturianas, Anleo, Spain). All the experimental tests were performed on at least two replicates of each type of sample. The liquid samples included: raw (RWM) and pasteurized (PWM) whole milk, pasteurized skim milk (PSKM), raw (RC) and pasteurized (PC) cream, raw (BM1) and pasteurized (BM2) buttermilk, and two samples of butterserum from butteroil (BS1 and BS2). These liquid samples were freeze-dried and stored at −35 °C. The powdered samples studied were: buttermilk powder (BMP-1), butterserum powder (BSP-1), and a mixture of buttermilk and butterserum powders (BM-BS BLEND). Dichloromethane, ethanol, chloroform, hexane, methanol, isooctane, isopropyl alcohol, and dimethylformamide were HPLC-grade and purchased from Carlo Erba (Val de Reuil, France). Sodium sulfate anhydrous was obtained from Panreac (Barcelona, Spain). Methyl tert-buthyl ether (MTBE) was supplied by VWR International Eurolab S.L. (Barcelona, Spain). Sodium methoxide (95%), sodium citrate dihydrate, formic acid (98%), acetic acid, and tritridecanoin (2,3-di(tridecanoyloxy)propyl tridecanoate) were supplied by Sigma-Aldrich (St. Louis, MO, USA).

### 2.2. Isolation of MFGM Fraction

Isolation of the MFGM fraction was performed using the method described by Corredig and Dalgleish [28] with some modifications as described by Calvo et al. [16]. Samples were reconstituted in Milli-Q water 10% (*w*/*v*) and stirred for 15 min at room temperature. Afterwards, 2% (*w*/*v*) of sodium citrate dihydrate was added to the mixture and incubated for 1 h at room temperature under constant stirring prior to ultracentrifugation at 70.000× *g* for 45 min at 15 °C in a Beckman Optima L-70 preparative ultracentrifuge (Beckman Coulter, Inc., Brea, CA, USA), then kept on ice for 30 min. Supernatant and the fat layer were carefully discarded and the MFGM pellet was frozen and stored as freeze-dried at −35 °C.

### 2.3. Analysis of Lipid Fraction

#### 2.3.1. Lipid Extraction

Fat was extracted using the simplified method described by Calvo et al. [16]. Briefly, 200 μL of sample (or 30 mg if powder, dissolved in 200 μL of Milli-Q) was mixed with 1.60 mL of methanol and 2.49 mL of dichloromethane and then stirred with a vortex for 20 min. Finally, acetic acid (1 mL of 20 mM) was added. The mixture was centrifuged at 3200 rpm for 5 min at 4 °C and the bottom organic phase was carefully removed. The upper methanol phase was washed again with dichloromethane, removed, and combined with that previously collected and passed through a syringe filter PVDF membrane with 0.45 μm pore size (Sigma-Aldrich) containing anhydrous sodium sulphate. Lipid extracts were collected in amber vials, flushed with nitrogen to dryness, weighted, and stored at −35 °C.

#### 2.3.2. Analysis of Lipid Classes by HPLC-ELSD

Separation of lipid classes was performed using an HPLC system (model 1260; Agilent Technologies Inc. Palo Alto, CA, USA) coupled with an ELSD (SEDEX 85 model; Sedere SAS, Alfortville Cedex, France) using pre-filtered compressed air as the nebulizing gas at a pressure of 350 kPa at 60 °C and the gain set at 3. Two columns in series (250 × 4.5 mm Zorbax Rx-SIL column with 5 μm particle diameter; Agilent Technologies Inc., Palo Alto, CA, USA) and a pre-column with the same packing were used. Before analysis, samples were dissolved in CH_2_Cl_2_ (at 5 mg/mL), and 50 μL was injected after column equilibration at 40 °C. The solvent gradient was as described by Castro-Gómez et al. [29]. Samples and standards were analyzed under the same conditions, using freshly prepared solvents. Assays were carried out in triplicate.

#### 2.3.3. Triacylglycerides and Cholesterol Determination Using GC-FID

Triacylglyceride (TAG) analysis was performed following Fontecha et al. (2005) [30], using a Clarus 400 GC (PerkinElmer Ltd., Beaconsfield, UK) equipped with an automatic split/splitless injector and a flame ionization detector. An Rtx-65TAG fused silica capillary column (30 m × 0.25 mm i.d. × 0.1 μm film thickness; Restek Corp., Bellefonte, PA) was used. Experimental chromatographic conditions were carried out with a temperature program as follows: 120 °C held for 30 s, 10 °C/min to 220°C held for 30 s, and 6 °C/min to 350 °C held for 30 min. Injector and flame ionization detector temperatures were 355 and 370 °C, respectively. Helium was used as the carrier gas (172 kPa) and the injection volume was 0.5 μL of dilutions of fat (30 mg/mL) in hexane. For qualitative and quantitative analysis of TAG, response factors were calculated using standard anhydrous milk fat at 20 mg/mL (reference material BCR-519; Fedelco Inc., Madrid, Spain) of known TAG and Chol composition.

#### 2.3.4. Determination of Fatty Acid Methyl Esters (FAMEs) Using GC-FID

FAMEs were prepared via direct transesterification using methanolic sodium methoxide (5% solution) according to the method described by Golay et al. (2006) [31]. GC-FID analysis was carried out on an Autosystem chromatograph (Perkin Elmer, Beaconsfield, UK) fitted with a VF-23 ms, fused silica capillary column (30 m × 0.25 mm i.d. × 0.25 μm film thickness, Varian, Middelburg, Netherlands) and FID. The column was held at 60 °C for 1 min after injection, temperature-programmed at 10 °C/min to 130 °C, then temperature-programmed at 3 °C/min to 170 °C, with the last ramp at 10 °C/min to 230 °C and being held there for 5 min. Helium was the carrier gas, with a column inlet pressure set at 20 psig and a split ratio of 1:20. The injection volume was 0.5 μL. Total run time was 32 min. The injector and detector temperatures were set at 250 °C and 270 °C, respectively. For qualitative and quantitative analysis, response factors were calculated using anhydrous milk fat (reference material BCR-164; Fedelco Inc., Madrid, Spain). Tritridecanoin (2,3-di(tridecanoyloxy)propyl tridecanoate; Sigma, St. Louis, MO, USA) was used as an internal standard (100 μL; 1.2 mg/mL) and added to lipid extracts before methylation. All the analyses were carried out in triplicate.

### 2.4. Protein Characterization

#### 2.4.1. Protein Content Quantification via Bradford Method

The protein concentrations of all samples were assessed through the Bradford assay, using the Pierce™ Coomassie (Bradford, UK) Protein Assay Kit (Thermo Fisher Scientific, Inc., Waltham, MA, USA). The assay was performed in 96-well microplates by mixing 10 µL of sample with 300 µL of Coomassie reagent per well (tests were made in triplicate). After 10 min of incubation at room temperature, absorbance was measured at 595 nm using the BioTek^®^ Cytation 5 Cell Imaging Multi-Mode Reader (Agilent Technologies, Inc., Santa Clara, CA, USA). Absorbance values were then interpolated onto the BSA standard curve to determine the protein concentration.

#### 2.4.2. Protein Identification via SDS-PAGE

The protein profile was investigated by means of sodium dodecyl sulfate-polyacrylamide gel electrophoresis (SDS-PAGE). Samples were normalized according to protein content (4 mg/mL) and then dissolved in sample buffer (4× XT Sample Buffer, Bio-Rad, Hercules, CA, USA) containing 5mL/100mL of Tris (2-carboxyethyl) phosphine hydrochloride (TCEP.HCl). The mix, with a final protein concentration of 1mg/mL, was heated at 95 °C for 3 min and then loaded on a 12% Bis-Trispolyacrilamide gel (Criterion_XT, Bio-Rad). Moreover, the protein pattern of MFGM isolated from selected BM and BS was investigated by means of 3–8% Tris-Acetate gels (Criterion_XT, Bio-Rad), which allowed for better separation and discrimination of high-molecular-weight proteins (50–250 kDa). Electrophoresis was conducted for 1 h at a constant voltage of 150 V. Finally, the 2-D gels were stained with a dye (Bio-Safe™ Coomassie G-250, Bio-Rad) and scanned with a Versa-Doc image system (Bio-Rad).

#### 2.4.3. Protein Identification via MALDI-TOF/TOF

Bands of interest were excised from the 1D gel and digested using an In-Gel Tryptic Digestion Kit (Thermo Fisher Scientific, Inc., Waltham, Massachusetts, USA). First, gel bands were destained with acetonitrile and 50 mM NH_4_HCO_3_ (1:1; *v*:*v*). Then, the cysteine residues of proteins were reduced using 0.5 M TCEP in 25 mM NH_4_HCO_3_ (1:10; *v*:*v*) and alkylated with a 100 mM iodoacetamide solution. Finally, gel pieces were dehydrated in 100% acetonitrile. The in-gel digestion was carried out at 37 °C, overnight by adding 10 μL of trypsin (89871B, Thermo Fisher Scientific) at a final concentration of 10 ng/μL in 25 mM NH_4_HCO_3_. The digested samples were spotted on a MALDI target plate together with α-cyano-4-hydroxycinnamic acid matrix (Thermo Fisher Scientific). The mass spectrometer used was an Autoflex SpeedTM (Bruker Daltonics GmbH & Co. KG, Bremen, Germany). Ions were detected in positive reflection mode at a mass range of *m*/*z* 600–3.500, and the laser pulses accumulated were 1.000 on average. Peptide calibration standard (Bruker Daltonics) was employed for external calibration of spectra. Monoisotopic peaks selected using the Flex Analysis 3.3 (Bruker Daltonics) software were fragmented using the lift method for MS/MS analysis. Peptide identification was performed using a home-made database of MFGM proteins running the MASCOT v2.4 software (Matrix Science, Inc., Chicago, IL, USA) Server 2.1 coupled with Biotools version 2.1 (Bruker Daltonics).

### 2.5. Statistical Analysis

Statistical analyses were performed using the SPSS package (SPSS 27.0 for Windows, SPSS Inc., Chicago, IL, USA). Differences among sample types were determined by performing one-way analysis of variance (ANOVA) with Duncan’s multiple range tests and *p* < 0.05 was accepted as significant value. Results are expressed as mean value ± standard deviation of at least two replicates.

## 3. Results and Discussion

### 3.1. Analysis of Lipid Classes and Protein Characterization of Raw Materials and By-Products of Butter and Butter Oil Processing

The fat content of the samples under investigation is shown in Table 1. As expected, fat content greatly differed among the samples depending on the manufacturing stage, from 1% to 88% for PSKM and RC, respectively. Among the by-products, the highest fat content (*p* < 0.05) corresponded to BS2 with a value of 27%, while BSP-1 featured the lowest. The distribution between neutral lipid (NL) and polar lipid (PL) contents was very different among the samples due to their different fat content and the technological fractionation process used (Table 1). Thus, the highest NL content was found in the creams (around 87%) due to their high triacylglycerol (TAG) content. On the contrary, after skimming, churning, and concentration processes, PSKM and by-products demonstrated lower fat contents (lower TAG concentration) but higher PL contents than the other types of sample. The highest PL values were found in BM1, BMP-1, and BS2 by-products (around 3%), slightly higher than those reported by Costa et al. [32]. Other authors, following a similar experimental design, also found a greater PL content in BS samples (up to 11.5%) [33]. Finally, although slight variations in NL and PL content were found among the different samples of butter and butter oil production (see flow chart, Figure 1), they were statistically significant only in some cases, especially when a heat treatment was applied. Since samples with a high fat content and a high polar lipid (PL) content are usually related to samples rich in MFGM, these data allowed us to select the most suitable raw materials and by-products for MFGM isolation and purification. Thus, the results of NL and PL content indicate that samples BM1, BMP-1, BS2, and BM-BS BLEND could be the most suitable for the isolation and purification of MFGM due to their higher PL/NL ratios.

The protein concentrations of milk (RWM, PWM, PSKM), cream (RC, PC), and the by-products of butter and butteroil manufacturing ranged from 3.4% to 35.3% (Table 1). As expected, raw and pasteurized cream (RC and PC) contained the lowest amount of protein (*p* < 0.05). In fact, they are classified as the dairy products with the highest fat content (Codex Alimentarius Commission, 2003) [34] at the expense of other macronutrients such as protein. On the contrary, protein content was significantly higher (*p* < 0.05) in pasteurized skim milk (PSKM), because the removal of fat causes an increase in protein concentration compared to whole milk (RWM and PWM). These results agree with Pugliese et al. (2017) [35], who reported an increase in protein content due to skimming. Furthermore, by-products from butter and butter oil production (BM1, BM2, BS1, BS2, BMP-1, BSP-1, BSP-1, BM-BS BLEND) were characterized by intermediate protein contents from 21% to 29%, which were likewise in the ranges of previously reported data [19,36].

### 3.2. Analysis of Fatty acid Composition

The fatty acid compositions of each product obtained at each stage of butter and butter oil processing is shown in Table 2. The main fatty acids found were palmitic acid (C16:0) and oleic acid (C18:1), followed in importance by myristic (C14:0) and stearic (C18:0) acids, which corresponds to that previously described extensively by other authors [37]. Saturated fatty acids (SFAs) were the major group in all the samples studied, with values ranging from 52% to 67%, followed by monounsaturated fatty acids (MUFAs) from 29% to 35%, and polyunsaturated fatty acids (PUFAs) from 4% to 9%. Significant lower SFA contents (*p* < 0.05) were found in the by-products (BM1, BMP-1 and BM-BS BLEND) of the butter making. These findings agree with those of other authors, being supported by the fact that the greater levels of MFGM of these samples are associated with higher levels of PL and, consequently, PUFA and MUFA [38]. However, this was not observed in butter oil by-products, particularly BS1 and BSP-1, which had similar SFA concentrations to whole milk. This appears to be related to the greater sphingomyelin (SM) content (see later, Section 3.5.2) and the significant occurrence of long-chain SFA (C16:0 and C18:0) in their structure [39]. Among the samples analyzed, the highest MUFA and PUFA contents were found in the BM1 and BM-BS BLEND buttermilks, where the value of oleic acid (C18:1 c9) was approximately 29% and that of linoleic acid (C18:2 c9,c12 n6) was around 6%. In addition, it was observed that the n6/n3 ratio was also higher in BM1, BS2, and BM-BS BLEND by-products. Although fatty acid composition does not seem to be significantly affected by the pasteurization process, the spray-drying heat treatment caused significant decreases in SFA in sample BMP-1 compared to the untreated BM2. In addition, the fatty acid compositions of BM and BS were different, with lower SFA contents in BM1 and BMP-1. Lopez et al. (2017) found differences between the fatty acids of polar lipids of BM and BS. According to these authors, BS has a higher SFA content compared to BM. Furthermore, they suggest that cream churning (during butter production) may retain the SFA-containing glycerophospholipids in butter and, therefore, BS would have a lower content [25]. Finally, the enhanced fatty acid profile of the samples BM1, BS2, BMP-1, and BM-BS BLEND guarantees that they are the most suitable for the isolation and purification of MFGM.

### 3.3. Triacylglycerols Determination

The triglycerol profiles of the different samples of milk, cream, and by-products of butter processing are shown in Table 3. Different triglyceride groups were identified according to the number of carbons, ranging from CN24 to CN54. TAG distribution corresponded to that described by Calvo et al., (2020) [16]. Two TAG peaks were found between CN36-CN40 and CN50. The content of low-molecular-weight TAG (LMW-TAG), from CN24 to CN34, exhibited significant differences between the samples. While the lowest contents were found in the milk and cream samples, the highest values were found in PSKM and by-products BMP-1 and BM-BS BLEND. These results can be linked to the co-elution of monoacylglycerides (MAG), diacylglycerols (DAG), and PL with the LMW-TAGs, as PSKM and by-products have lower TAG contents [40]. Furthermore, the major TAG was CN38, which is found in medium-molecular-weight TAGs (MMW-TAG). The content of this TAG was higher in the milk and cream samples and was related, as described above, with a higher content of palmitic acid (C16:0), while the lowest contents were achieved in PSKM, BM1, BS1, BMP-1, and BSP-1. As expected, milks and creams, in addition to some by-products (BM2, BS1, BSP-1), exhibited the highest content of high-molecular-weight TAGs (HMW-TAGs), while the highest value was observed in BMP-1.

### 3.4. Protein Identification via SDS-PAGE

**Figure 2:**
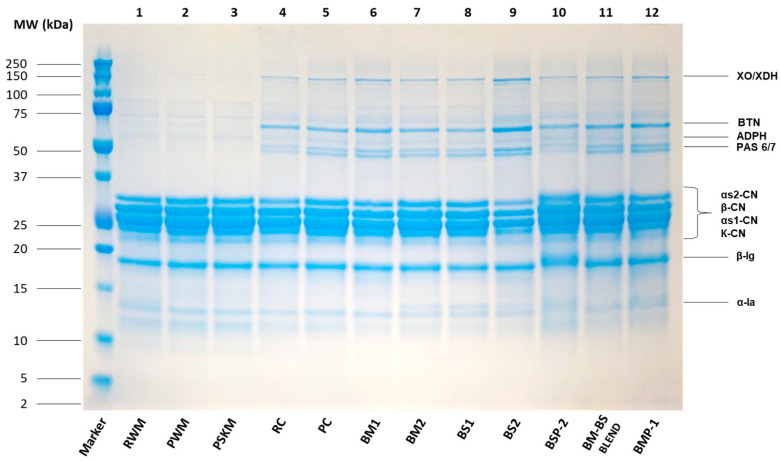
SDS-PAGE protein profile of the milk, cream, and by-product samples of the butter and butter oil manufacturing process. XDH/XO: xanthine dehydrogenase/oxidase, BTN: butyrophilin, ADPH: adipophilin, PAS 6/7: glycosylation variants of lactadherin, CN: casein, lg: lactoglobulin, la: lactalbumin. Markers: left column denotes the molecular weight markers (kDa). RWM: raw whole milk, PWM: pasteurized whole milk, PSKM: pasteurized skimmed milk, RC: raw cream, PC: pasteurized cream, BM1: buttermilk 1, BM2: buttermilk 2, BS1: butterserum 1, BS2: butterserum 2, BMP-1: buttermilk powder, BSP-1: butterserum powder, BM-BS BLEND: buttermilk and butterserum mix.

The patterns of proteins in milk (RWM, PWM, PSKM), cream (RC, PC), and by-products of butter and butteroil production (BM1, BM2, BS1, BS2, BMP-1, BSP-1, BM-BS BLEND) are shown on representative gels (Figure 2). Gel bands were putatively grouped based on MW as MFGM protein (45–150 kDa), casein (25–35 kDa), and whey protein (14–20 kDa). SDS-PAGE found important differences among samples, both qualitative and quantitative, since higher relative intensities denote major protein solubility [2]. Regarding the MFGM proteins, the most visible bands were associated with xanthine dehydrogenase/oxidase (XDH/XO), butyrophilin (BTN), adipophilin (ADPH), and the two glycosylation variants of lactadherin (PAS-6 and PAS-7). Recent studies have reported various health-enhancing properties associated with these proteins. Among the known beneficial effects are positive impacts on neurodevelopment, the cognitive and immune systems, and gut microbiota and functionality [13]. Milk samples (RWM, PWM, and PSKM) exhibited brighter bands compared to the other samples, thereby suggesting a lower concentration in MFGM proteins. On the contrary, the other samples exhibited more available MFGM proteins, probably because of some process operations (e.g., cream churning in butter processing) that promote the breakdown of fat globules and thus the release of membrane fragments [41,42]. In detail, the darkest bands were observed in samples BM1, BM2, BS2, BMP-1 and BM-BS BLEND, which were therefore selected as starting materials for MFGM isolation via ultracentrifugation. The casein fraction (αs1-, αs2-, β-, K- casein) and whey proteins (α-lactalbumin and β-lactoglobulin) were always clearly visible, with minor differences between samples.

### 3.5. Lipid and Protein Characterization of MFGM Isolates

Based on the findings mentioned in the preceding sections, the following by-products BM1, BM2, BS2, BMP-1, and BM-BS BLEND were chosen as the most suitable samples for the isolation and characterization of MFGM.

#### 3.5.1. Fat and Protein Content of MFGM Isolates

**Figure 3:**
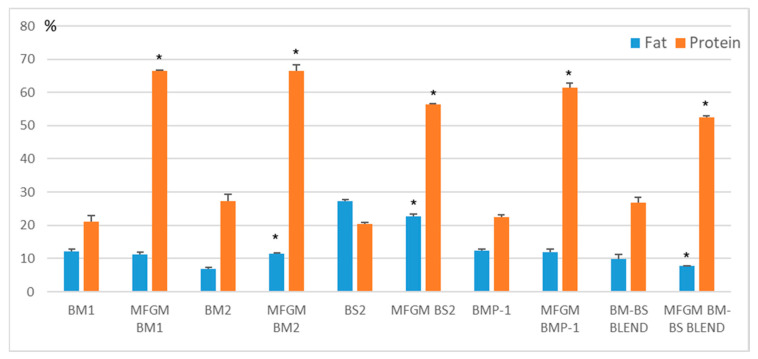
Fat and protein contents expressed as means of the selected samples and their corresponding MFGM isolates (g/100 g of sample ± standard deviation) of at least two replicates. *: in the column between each sample and their corresponding MFGM isolate, indicates significant differences (*p* < 0.05).

Figure 2 shows the fat and protein content of MFGM isolated from selected samples (BM1, BM2, BS2, BMP-1, and BM-BS BLEND). Ultracentrifugation did not affect fat content in the cases of BM1 and BMP-1, while it led to a significant reduction (*p* < 0.05) in BS2 and BM-BS BLEND. On the other hand, MFGM isolated from BM2 exhibited a higher fat amount compared to the starting sample BM2. Regarding the protein content, ultracentrifugation always led to a marked increase in this parameter (approximately three-fold). Generally, MFGM proteins make up 25–60% of the total MFGM mass, 1–4% of total milk proteins, and 1% of the total globule mass [5]. Our results were consistent with those indicated in the literature, varying from 53 to 66% (Figure 3). In detail, no statistical differences (*p* > 0.05) could be highlighted between the MFGM isolated from BM1 and BM2, which exhibited the highest protein concentration (*p* < 0.05) compared with the other samples, while the lowest value (*p* < 0.05) was found for the MFGM obtained from BM-BS BLEND (52.6%). These outcomes reasonably suggest that the yield of MFGM proteins may be greatly impaired by some treatments and processing steps applied throughout the butter/butter-oil production (e.g., heating, cooling, agitation, homogenization, evaporation, spray-drying). For example, there is ample evidence that whey proteins and immunoglobulins are sensitive to heat because of their globular structure that is prone to thermal unfolding [43]. MFGM proteins are also quite sensitive to temperature. In fact, recent studies revealed that heat treatments such as milk pasteurization (80 °C for 3 min) and ultra-heat treatment (125–145 °C) may significantly reduce the amounts of some native MFGM proteins (e.g., BTN, XDH/XOs and PAS 6/7) [44,45].

#### 3.5.2. Analysis of Lipid Classes of the Selected Samples and Their Corresponding MFGM Isolates

The lipid classes, NL and PL, of the MFGM fractions obtained via ultracentrifugation from the BM1, BM2, BS2, BMP-1, and BM-BS BLEND samples were analyzed. The different buttermilks were compared with their corresponding MFGM isolates (Table 4). As expected, TAG content was significantly lower in all MFGM isolates due to the separation of these lipids in the triacylglycerol fraction after ultracentrifugation. Therefore, the total NL content was significantly lower in all MFGM isolates. A better separation of the TAG fraction was achieved for MFGM-BM1 and MFGM-BM2 due to the lower TAG amounts, 25.8% and 29.3%, respectively. TAG contents of MFGM isolates were lower than the ones reported in the literature (56% TAG over total fat in MFGM) [46]. In contrast, the DAG content significantly increased in some MFGM isolates, including the MFGM-BM2, MFGM-BS2, and MFGM-BM-BS BLEND samples. The increase in DAG in these isolates may be due to the hydrolysis of the TAG fraction during processing. However, no differences were observed among MFGM-BM1 and MFGM-BMP-1 samples. The free fatty acid and cholesterol (FFA + Chol) content also significantly increased in MFGM isolates MFGM-BM2 and MFGM-BS2, but no differences were found in the other MFGM isolates. The increase in FFA + Chol is due to the high cholesterol content of the MFGM structure. Concerning MAG content, there were no differences between the by-products and their isolates, except for MFGM-BM2, which had a higher MAG content than BM2. Glucosylceramide (GlucCer) and Lactosylceramide (LacCer) contents increased in all MFGM isolates, since these compounds are associated with MFGM. As for the PL content, a two to seven-fold increase was achieved in all MFGM isolates compared with the relative buttermilks. Most of the MFGM isolates had a similar PL content to that described by other authors [16,47] who isolated MFGM from buttermilk (around 40% fat). In addition, the highest PL content was obtained for MFGM-BM1 isolate (55.7% fat).

**Figure 4:**
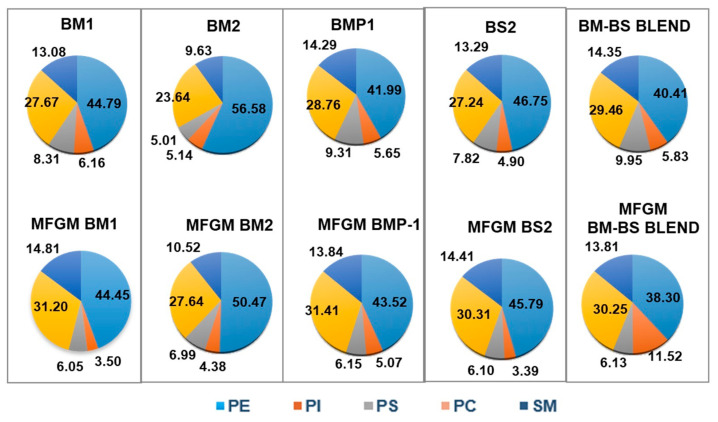
Distribution of phospholipids and sphingomyelin (mean values expressed as % of PL) of the selected samples and their corresponding MFGM isolates. PE: phosphatidylethanolamine, PC: phosphatidylcholine, PS: phoshatidylserine, PI: phosphatidylinositol, and SM: sphingomyelin of the buttermilk (BM) and butterserum (BS) and their corresponding MFGM isolates.

The distribution of PLs expressed as percentage of PL in the selected samples and their corresponding isolates is shown in Figure 4. The majority of PLs were phosphatidylethanolamine (PE) and phosphatidylcholine (PC), in agreement with other authors [48]. PE content increased significantly in all MFGM isolates, with higher percentages in MFGM-BM1 and MFGM-BMP-1, but its distribution did not change in most isolates, except MFGM-BM2, which exhibited a lower PE content with respect to BM2. The highest PC contents were obtained in MFGM isolates MFGM-BM1 and MFGM-BMP-1. With regard to phosphatidylinositol (PI), there were significant differences among all samples compared to their MFGM isolates, with most of the isolates presenting a lower PI content. However, it is noteworthy that the highest PI value was achieved in MFGM-BM-BS BLEND. On the other hand, phosphatidylserine (PS) content also significantly increased in all MFGM isolates except MFGM-BMP-1. In addition, PS distribution varied greatly between the samples and the corresponding MFGM isolates, with lower PS contents for the MFGM isolates, except in the case of MFGM-BM2. As expected, the sphingomyelin (SM) content also increased in MFGM isolates, with the highest value obtained in MFGM-BM1.

#### 3.5.3. Fatty Acid Methyl Esters Analysis of the Selected Samples and Their Corresponding MFGM Isolates

**Figure 5:**
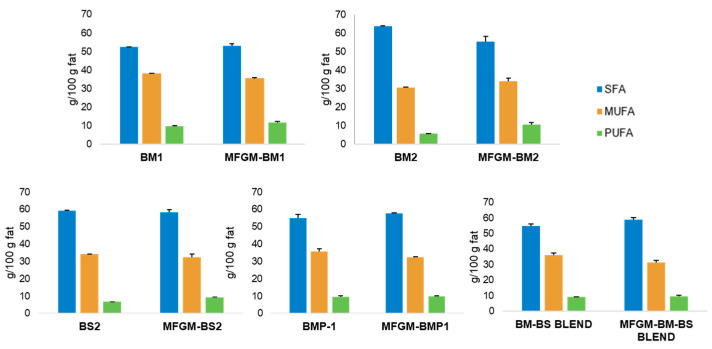
Distribution of saturated fatty acid (SFA), monounsaturated fatty acid (MUFA), and polyunsaturated fatty acid (PUFA) content (values expressed as g/100g of fat ± standard derivation) of the selected samples and their corresponding MFGM isolates.

The SFA, MUFA, and PUFA contents of the MFGM isolates are shown in Figure 5. SFA content in MFGM isolates ranged from 53 to 58.9%, indicating a higher purity in comparison with the outcomes of other authors such as Lu et al. (2016) [49], who reported SFA values between 71.7% and 72.5%. On the other hand, it was observed that the MUFA content ranged from 31.4% to 35.4% and PUFA content ranged from 9.3% to 11.8%. Each MFGM isolate was compared with the corresponding by-product. Some MFGM isolates exhibited significant differences in PUFA and MUFA content. The highest PUFA contents were found in MFGM-BM1, MFGM-BM2, and MFGM-BS2 due to the removal of the TAG fraction containing the highest SFA content. Calvo et al. [16] also described an increase in PUFA in MFGM isolates of buttermilk origin. This increase in PUFA is the result of an increase in PL in this fraction. The MUFA content decreased significantly in MFGM-BM1, MFGM-BMP-1, and MFGM-BM-BS BLEND due to an increase in PUFA. However, the MUFA content increased in MFGM-BM2 because BM2 contained a high level of SFA, which decreased in the corresponding isolate. In addition, SFA content increased in MFGM-BMP-1 and MFGM BM-BS BLEND, which could be due to the increase in PL and, thus, SM with high presence of SFA of long chain, as previously indicated.

#### 3.5.4. Triacylglyceride Determination of the Selected Samples and Their Corresponding MFGM Isolates

**Figure 6:**
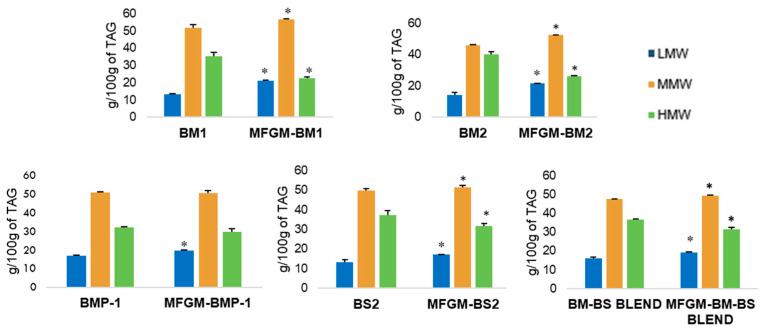
TAG profile of the low-molecular-weight LMW-TAG (CN26-CN34), medium-molecular-weight MMW-TAG (CN36-CN44), and high-molecular-weight HMW-TAG (CN46-CN-54) of the selected samples and their corresponding MFGM isolates. The data are expressed as means (g/100 g of fat ± standard deviation) of at least two replicates. * indicates significant differences (*p* < 0.05).

The TAG concentration of the selected samples is shown in Figure 6. A significant increase in LMW-TAG content was observed in all MFGM isolates, which was due to an increase in DAG, MAG, and PL content, as described in Section 3.5.2. Calvo et al. [16] also reported an increase in LMW-TAG content in MFGM isolates. As for the MMW-TAG content, an increase was also observed in MFGM-BM1, MFGM-BM2, and the MFGM-BM-BS BLEND isolates. This resulted in a proportional decrease in the HMW-TAG fraction, which may be linked to TAG removal during the MFGM isolation process and the consequent increase in PL in these MFGM isolates. These results are also in agreement with those described by Calvo et al. [16].

#### 3.5.5. Protein Characterization of the Selected Samples and Their Corresponding MFGM Isolates

**Figure 7:**
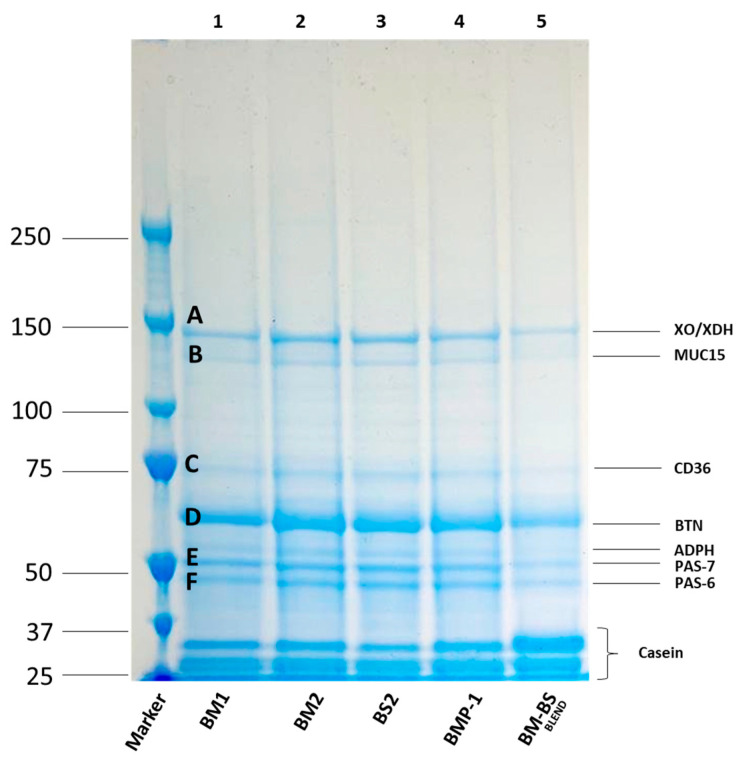
SDS-PAGE pattern of MFGM isolated from selected samples. XDH/XO: xanthine dehydrogenase/oxidase; MUC15: mucin; CD36: cluster of differentiation; BTN: butyrophilin; ADPH: adipophilin; PAS 6/7: glycosylation variants of lactadherin, marker: left column denotes the molecular weight markers (kDa). BM1: buttermilk 1, BM2: buttermilk 2, BS2: butter-serum 2, BMP-1: buttermilk powder, BM-BS BLEND: buttermilk and butterserum mix.

SDS-PAGE protein profiles of MFGMs isolated from selected samples are depicted in Figure 7. In this case, 3–8% Tris-Acetate gels (Criterion_XT, Bio-Rad) were used to better separate and discriminate high-molecular-weight proteins (50–250 kDa). Recent proteomic studies surveyed 20 to 411 MFGM proteins unique for bovine milk [50,51,52,53,54]. The huge differences found in the number of proteins identified may be attributed to several variables, such as intrinsic factors (lactation stage and breed), sample treatments, MFGM isolation techniques, and proteomic methods applied (e.g., SDS-PAGE, 2-D electrophoresis, MALDI-TOF-MS, LC-Orbitrap MS/MS, EASY-nLC-Orbitrap MS/MS) [55]. Among the latter, the assessment of stained electrophoretic band intensity has been extensively used [56,57,58]. Overall, MFGMs isolated from BM-BS BLEND (line 5) demonstrated lower band relative intensities compared to the other samples. These findings could be reasonably associated with the severe conditions applied during spray-drying and evaporation processes. In fact, MFGMs are highly susceptible to rapid air beating, intense turbulence, high velocity gradient, and temperature [59]. The last item in particular may hugely impair the stability and the integrity of the MFGM, inducing MFGM protein denaturation and binding with whey proteins above 60 °C [23]. In addition, a large proportion of caseins could be seen at lower molecular weight (≈25 kDa). Morin et al. [60] faced the same issue, justifying the presence of these caseins with their trapping and dragging by MFGMs during MFGM isolation via ultracentrifugation. Furthermore, a recent investigation by Hansen et al. [61] found that heat treatment (such as pasteurization) not only increased the interaction between casein and MFGM proteins but also that this association was higher with increasing heating steps. As listed in Table 5, the spectrometric analysis of MFGM proteins via MALDI-TOF/TOF (Figure 7) led to the recognition of four different MFGM proteins (A, D, E, and F) which correspond to XDH/XO (146.8 kDa), BTN (59.3 kDa), PAS-6 (47 kDa), and PAS-7 (50 kDa). In addition, the B and C bands matched MUCIN15 and CD36, respectively. These outcomes agree with Pallesen et al. [62], who stated that the gel band at ≈ 130 kDa may be associated with the mucin MUC15, a highly glycosylated protein originally described as a high-molecular-weight glycoprotein (PAS III). Indeed, CD36 is an integral protein with an apparent molecular weight within 75–80 kDa on SDS gels [63].

## 4. Conclusions

In this work, dairy products and by-products generated by the butter and butter oil manufacturing process were studied to comparatively determine their potential as profitable sources of MFGM. By combining lipidomics and proteomics, we worked out a detailed characterization of the components present in the different by-products that are currently available. Namely, we elucidated the patterns of total (neutral and polar) lipids and proteins (particularly MFGM proteins) of the isolated MFGM fractions as well as the fluctuations that follow the various technological and thermal processes used. Because MFGM are associated (to a greater or lesser extent) with TAG from the milk fat globule, and whey proteins and caseins are also adsorbed to MFGM, it was possible to discriminate those by-products in which the proportion of polar lipids and membrane proteins was higher. These results allow us to conclude that the BM1, BMP-1, BS2, and BM-BS BLEND samples could be advantageously employed for the isolation and purification of bioactive MFGM as the result of having the highest PL/NL ratio as well as a high content of membrane proteins.

Following future, indispensable studies to determine the bioaccessibility and bioavailability of MFGM isolates obtained from butter and butter oil by-products and an appropriate industrial scale-up, we envision the use of currently underutilized food waste to prepare MFGM-based nutraceuticals and functional foods to be potentially employed in the medical food and human health area [64]. RWM: raw whole milk, PWM: pasteurized whole milk, PSKM: pasteurized skimmed milk, RC: raw cream, PC: pasteurized cream, BM1: buttermilk 1, BM2: buttermilk 2, BS1: butterserum 1, BS2: butterserum 2, BMP-1: buttermilk powder, BSP-1: butterserum powder, BM-BS BLEND: buttermilk and butterserum mix.

## Figures and Tables

**Table 1:** Mean values of the fat, neutral lipid (NL), polar lipid (PL), and protein of the milk, cream, and by-product samples of the butter and butter oil manufacturing process.

Sample	Fat	NL	PL	Protein
RWM	29.63 ± 4.85 ^d^	29.63 ± 4.85 ^e^	n.d.	24.10 ± 0.62 ^de^
PWM	34.04 ± 0.76 ^e^	34.04 ± 0.77 ^f^	n.d.	19.43 ± 0.48 ^b^
PSKM	1.07 ± 0.52 ^a^	0.91 ± 0.43 ^a^	0.16 ± 0.09 ^a^	35.28 ± 0.74 ^i^
RC	88.69 ± 1.07 ^g^	88.69 ± 1.08 ^h^	n.d.	3.60 ± 0.25 ^a^
PC	85.38 ± 1.25 ^f^	85.38 ± 1.26 ^g^	n.d.	3.46 ± 0.36 ^a^
BM1	12.18 ± 0.61 ^c^	8.80 ± 0.51 ^c^	3.38 ± 0.10 ^e^	21.07 ± 1.77 ^bc^
BM2	6.91 ± 0.52 ^b^	6.52 ± 0.50 ^bc^	0.38 ± 0.02 ^b^	27.35 ± 1.98 ^g^
BS1	6.18 ± 1.22 ^b^	5.66 ± 1.12 ^b^	0.36 ± 0.15 ^b^	29.56 ± 1.47 ^h^
BS2	27.28 ± 0.44 ^d^	24.25 ± 0.37 ^d^	3.02 ± 0.07 ^d^	20.48 ± 0.42 ^b^
BMP-1	12.28 ± 0.51 ^c^	8.91 ± 0.32 ^c^	3.37 ± 0.19 ^e^	22.52 ± 0.70 ^cd^
BSP-1	5.51 ± 0.83 ^b^	5.08 ± 0.75 ^b^	0.43 ± 0.09 ^b^	25.24 ± 1.33 ^ef^
BM-BS BLEND	9.91 ± 1.24 ^c^	8.01 ± 1.14 ^bc^	1.91 ± 0.13 ^c^	26.84 ± 1.58 ^fg^

The data are expressed as means (g/100 g ± standard deviation) of at least two replicates. The different superscripts in the same column indicate significant differences (*p* < 0.05). n.d.: not detectable.

**Table 2:** Mean values of fatty acid methyl esters (FAMEs) of the milk, cream and by-product samples of the butter and butter oil manufacturing process.

FAME	RWM	PWM	PSKM	RC	PC	BM1	BM2	BS1	BS2	BMP-1	BSP-1	BM-BS BLEND
C4:0	4.82 ± 1.18 ^abc^	4.57 ± 0.31 ^ab^	6.61 ± 1.41 ^cd^	5.65 ± 0.28 ^abc^	5.59 ± 0.94 ^abc^	4.30 ± 0.09 ^ab^	6.26 ± 0.15 ^bcd^	4.63 ± 0.28 ^abc^	5.12 ± 0.82 ^abc^	4.04 ± 0.56 ^a^	7.72 ± 2.71 ^d^	4.06 ± 0.42 ^a^
C6:0	2.86 ± 0.60 ^e^	2.50 ± 0.24 ^de^	2.25 ± 0.48 ^cde^	2.68 ± 0.40 ^de^	2.73 ± 0.34 ^e^	1.30 ± 0.10 ^a^	2.77 ± 0.01 ^e^	1.65 ± 0.14 ^abc^	2.02 ± 0.02 ^bcd^	1.56 ± 0.14 ^ab^	2.29 ± 0.85 ^cde^	1.46 ± 0.11 ^ab^
C8:0	2.09 ± 0.42 ^e^	1.86 ± 0.21 ^de^	1.45 ± 0.15 ^bc^	1.98 ± 0.30 ^de^	1.88 ± 0.14 ^de^	0.92 ± 0.10 ^a^	2.02 ± 0.18 ^e^	1.31 ± 0.03 ^abc^	1.60 ± 0.09 ^cd^	1.27 ± 0.15 ^abc^	1.57 ± 0.38 ^cd^	1.06 ± 0.12^ab^
C10:0	4.43 ± 0.68 ^ef^	4.11 ± 0.33 ^def^	3.22 ± 0.49 ^abc^	4.18 ± 0.46 ^ef^	4.22 ± 0.51 ^ef^	2.40 ± 0.03 ^a^	4.72 ± 0.29 ^f^	3.25 ± 0.30 ^abcd^	3.73 ± 0.23 ^bcde^	2.95 ± 0.28 ^ab^	4.08 ± 1.07 ^cdef^	2.66 ± 0.08 ^a^
C12:0	4.61 ± 0.25 ^f^	4.48 ± 0.18 ^ef^	4.02 ± 0.23 ^de^	4.71 ± 0.25 ^f^	4.72 ± 0.35 ^f^	2.81 ± 0.01 ^a^	4.72 ± 0.02 ^f^	3.58 ± 0.08 ^cd^	3.91 ± 0.03 ^d^	3.35 ± 0.34 ^bc^	4.06 ± 0.62 ^de^	3.04 ± 0.06 ^ab^
C14:0	12.21 ± 0.16 ^ef^	12.40 ± 0.01 ^f^	13.08 ± 0.54 ^g^	12.50 ± 0.29 ^f^	12.35 ± 0.02 ^f^	9.03 ± 0.01 ^a^	11.79 ± 0.14 ^de^	11.81 ± 0.19 ^de^	10.61 ± 0.34 ^c^	9.56 ± 0.47 ^b^	11.40 ± 0.39 ^d^	9.70 ± 0.15 ^b^
C14:1 c9	1.15 ± 0.06 ^a^	1.15 ± 0.03 ^a^	1.13 ± 0.07 ^a^	1.18 ± 0.07 ^a^	1.30 ± 0.04 ^bc^	1.11 ± 0.04 ^a^	1.19 ± 0.07 ^ab^	1.31 ± 0.04 ^c^	1.08 ± 0.01 ^a^	1.14 ± 0.06 ^a^	1.30 ± 0.16 ^bc^	1.13 ± 0.03 ^a^
C16:0	23.50 ± 0.74 ^c^	23.51 ± 0.44 ^c^	23.08 ± 0.13 ^c^	23.68 ± 1.29 ^c^	23.63 ± 0.54 ^c^	20.07 ± 0.02 ^a^	20.75 ± 0.08 ^ab^	24.74 ± 0.03 ^d^	21.07 ± 0.46 ^ab^	20.39 ± 0.25 ^ab^	21.52 ± 1.31 ^b^	20.95 ± 0.06 ^ab^
C16:1 c9	1.15 ± 0.05 ^de^	1.13 ± 0.02 ^cde^	1.08 ± 0.08 ^bcd^	1.18 ± 0.04 ^e^	1.19 ± 0.01 ^e^	1.07 ± 0.01 ^abcd^	1.13 ± 0.06 ^cde^	0.99 ± 0.04 ^a^	1.06 ± 0.04 ^abc^	1.09 ± 0.04 ^bcd^	1.09 ± 0.09 ^bcd^	1.03 ± 0.02 ^ab^
C18:0	6.70 ± 0.24 ^bcd^	6.99 ± 0.17 ^cd^	6.56 ± 0.59 ^bc^	6.67 ± 0.30 ^bc^	6.29 ± 0.23 ^ab^	7.69 ± 0.02 ^e^	5.96 ± 0.02 ^a^	7.91 ± 0.02 ^e^	6.95 ± 0.07 ^cd^	7.77 ± 0.09 ^e^	7.18 ± 0.55 ^d^	7.66 ± 0.08 ^e^
C18:1 t10	0.24 ± 0.05 ^ab^	0.43 ± 0.08 ^c^	0.14 ± 0.14 ^a^	0.43 ± 0.07 ^c^	0.38 ± 0.06 ^c^	0.35 ± 0.06 ^bc^	0.24 ± 0.01 ^ab^	0.46 ± 0.01 ^c^	0.41 ± 0.01 ^c^	0.37 ± 0.11 ^c^	0.23 ± 0.04 ^a^	0.36 ± 0.01 ^c^
C18:1 t11 (TVA)	0.36 ± 0.06 ^a^	0.92 ± 0.31 ^b^	0.38 ± 0.38 ^a^	0.37 ± 0.37 ^a^	0.60 ± 0.10 ^ab^	0.54 ± 0.01 ^ab^	0.66 ± 0.06 ^ab^	0.53 ± 0.01 ^ab^	0.74 ± 0.16 ^ab^	0.63 ± 0.04 ^ab^	0.62 ± 0.26 ^ab^	0.61 ± 0.07 ^ab^
C18:1 c9	24.70 ± 1.68 ^a^	24.20 ± 0.30 ^a^	24.54 ± 0.45 ^a^	23.32 ± 1.10 ^a^	23.17 ± 1.06 ^a^	30.22 ± 0.23 ^c^	23.36 ± 0.33 ^a^	24.43 ± 0.04 ^a^	26.66 ± 0.85 ^b^	29.65 ± 1.16 ^c^	23.14 ± 2.66 ^a^	29.06 ± 0.75 ^c^
Total cis C18:1	25.84 ± 1.80 ^a^	25.47 ± 0.03 ^a^	24.99 ± 0.01 ^a^	24.54 ± 1.01 ^a^	24.53 ± 1.17 ^a^	32.38 ± 0.18 ^c^	24.99 ± 0.44 ^a^	25.90 ± 0.08 ^a^	28.33 ± 0.74 ^b^	30.65 ± 1.52 ^c^	24.22 ± 3.47 ^a^	30.75 ± 1.11 ^c^
Total trans C18:1	1.96 ± 0.21 ^a^	2.86 ± 0.62 ^ab^	1.97 ± 1.43 ^a^	1.96 ± 0.77 ^a^	2.60 ± 0.20 ^ab^	2.88 ± 0.02 ^ab^	2.70 ± 0.05 ^ab^	2.66 ± 0.08 ^ab^	3.09 ± 0.55^b^	2.38 ± 0.07 ^ab^	2.50 ± 0.69 ^ab^	2.75 ± 0.16 ^ab^
C18:2 c9.c12 (LA)	2.57 ± 0.19 ^a^	2.57 ± 0.12 ^a^	4.13 ± 0.36 ^cd^	2.49 ± 0.09 ^a^	2.41 ± 0.24 ^a^	6.85 ± 0.20 ^e^	3.50 ± 0.32 ^bc^	3.34 ± 1.05 ^b^	4.71 ± 0.02 ^d^	6.56 ± 0.66 ^e^	3.35 ± 0.22 ^b^	6.44 ± 0.04 ^e^
C18:3 (ALA)	0.40 ± 0.04 ^a^	0.54 ± 0.05 ^ab^	0.41 ± 0.41 ^a^	0.43 ± 0.08 ^a^	0.44 ± 0.02 ^ab^	0.61 ± 0.01 ^ab^	0.52 ± 0.08 ^ab^	0.46 ± 0.03 ^ab^	0.39 ± 0.09 ^a^	0.43 ± 0.16 ^a^	0.70 ± 0.06 ^b^	0.57 ± 0.03 ^ab^
C18:2 c9.t11 (RA)	0.70 ± 0.04 ^bcd^	0.91 ± 0.15 ^cd^	0.30 ± 0.31 ^a^	0.62 ± 0.01 ^bc^	0.52 ± 0.11 ^ab^	0.93 ± 0.10 ^d^	0.81 ± 0.26 ^cd^	0.90 ± 0.20 ^cd^	0.83 ± 0.02 ^cd^	1.24 ± 0.22 ^e^	0.72 ± 0.06 ^bcd^	0.88 ± 0.05 ^cd^
C20:3 n6 (DGLA)	0.09 ± 0.01 ^a^	0.08 ± 0.03 ^a^	0.03 ± 0.03 ^a^	0.01 ± 0.01 ^a^	0.07 ± 0.07 ^a^	0.71 ± 0.06 ^d^	0.44 ± 0.04 ^bc^	0.33 ± 0.07 ^b^	0.36 ± 0.12 ^b^	0.52 ± 0.12 ^c^	0.13 ± 0.13 ^a^	0.56 ± 0.04 ^c^
C20:4 n6 (AA)	0.11 ± 0.01 ^a^	0.10 ± 0.03 ^a^	0.15 ± 0.15 ^a^	0.06 ± 0.06 ^a^	0.13 ± 0.01 ^a^	0.66 ± 0.07 ^e^	0.45 ± 0.08 ^bc^	0.37 ± 0.05 ^b^	0.50 ± 0.03 ^cd^	0.67 ± 0.07 ^e^	0.07 ± 0.07 ^a^	0.60 ± 0.01 ^de^
Total n3	0.40 ± 0.04 ^a^	0.54 ± 0.05 ^ab^	0.41 ± 0.41 ^a^	0.43 ± 0.08 ^a^	0.44 ± 0.02 ^ab^	0.61 ± 0.01 ^ab^	0.52 ± 0.08 ^ab^	0.46 ± 0.03 ^ab^	0.39 ± 0.09 ^a^	0.43 ± 0.16 ^a^	0.70 ± 0.06 ^b^	0.57 ± 0.03 ^ab^
Total n6	2.78 ± 0.19 ^ab^	2.75 ± 0.17 ^ab^	4.31 ± 0.18 ^c^	2.56 ± 0.16 ^a^	2.61 ± 0.30 ^a^	8.21 ± 0.32 ^e^	4.39 ± 0.36 ^c^	4.03 ± 1.16 ^c^	5.58 ± 0.11 ^d^	7.76 ± 0.83 ^e^	3.55 ± 0.42 ^bc^	7.61 ± 0.09 ^e^
n6/n3	6.98 ± 0.29 ^b^	5.16 ± 0.76 ^ab^	2.54 ± 2.54 ^a^	6.12 ± 0.66 ^ab^	5.91 ± 0.48 ^ab^	13.44 ± 0.76 ^c^	8.68 ± 1.95 ^b^	8.88 ± 3.06 ^b^	15.07 ± 2.96 ^c^	19.91 ± 5.37 ^d^	5.13 ± 1.05 ^ab^	13.42 ± 0.52 ^c^

The data are expressed as means (g/100 g of fat ± standard deviation) of at least two replicates. Different superscripts in the same row indicate significant differences (*p* < 0.05). RWM: raw whole milk, PWM: pasteurized whole milk, PSKM: pasteurized skimmed milk, RC: raw cream, PC: pasteurized cream, BM1: buttermilk 1, BM2: buttermilk 2, BS1: butterserum 1, BS2: butterserum 2, BMP-1: buttermilk powder, BSP-1: butterserum powder, BM-BS BLEND: buttermilk and butterserum mix.

**Table 3:** Mean values of triacylglycerol (TAG) content of milk, cream and by-product samples of the butter and butter oil manufacturing process.

TAG (%)	RWM	PWM	PSKM	RC	PC	BM1	BM2	BS1	BS2	BMP-1	BSP-1	BM-BS BLEND
CN24	0.08 ± 0.02 ^ab^	0.08 ± 0.03 ^ab^	0.50 ± 0.21 ^e^	0.05 ± 0.02 ^a^	0.05 ± 0.02 ^a^	0.31 ± 0.13 ^d^	0.17 ± 0.09 ^abcd^	0.29 ± 0.05 ^cd^	0.22 ± 0.01 ^bcd^	0.14 ± 0.03 ^abc^	0.24 ± 0.01 ^cd^	0.17 ± 0.06 ^abcd^
CN26	0.33 ± 0.02 ^a^	0.32 ± 0.02 ^a^	1.08 ± 0.46 ^c^	0.27 ± 0.06 ^a^	0.28 ± 0.02 ^a^	0.48 ± 0.15 ^a^	0.55 ± 0.34 ^a^	0.55 ± 0.07 ^a^	0.67 ± 0.17 ^ab^	0.97 ± 0.27 ^bc^	0.63 ± 0.27 ^ab^	0.98 ± 0.05 ^bc^
CN28	0.61 ± 0.01 ^c^	0.63 ± 0.04 ^c^	0.54 ± 0.03 ^b^	0.64 ± 0.01 ^c^	0.64 ± 0.01 ^c^	0.63 ± 0.01 ^c^	0.72 ± 0.04 ^d^	0.46 ± 0.03 ^a^	0.66 ± 0.06 ^c^	0.81 ± 0.01 ^e^	0.47 ± 0.06 ^a^	0.72 ± 0.02 ^d^
CN30	1.32 ± 0.01 ^a^	1.35 ± 0.06 ^a^	2.14 ± 0.09 ^d^	1.33 ± 0.01 ^a^	1.36 ± 0.01 ^a^	1.66 ± 0.04 ^bc^	1.53 ± 0.07 ^ab^	1.29 ± 0.49 ^a^	1.66 ± 0.20 ^bc^	2.18 ± 0.01 ^d^	1.58 ± 0.07 ^ab^	1.92 ± 0.07 ^cd^
CN32	2.73 ± 0.01 ^a^	2.76 ± 0.13 ^a^	4.53 ± 0.39 ^c^	2.73 ± 0.03 ^a^	2.79 ± 0.08 ^a^	3.47 ± 0.24 ^b^	3.67 ± 0.41 ^b^	3.34 ± 0.62 ^b^	3.30 ± 0.36 ^b^	4.57 ± 0.26 ^c^	3.66 ± 0.29 ^b^	4.25 ± 0.19 ^c^
CN34	6.10 ± 0.05 ^a^	6.24 ± 0.16 ^a^	7.73 ± 0.34 ^bc^	6.17 ± 0.09 ^a^	6.22 ± 0.07 ^a^	6.49 ± 0.44 ^a^	7.41 ± 0.75 ^b^	7.85 ± 0.40 ^bc^	6.57 ± 0.74 ^a^	8.27 ± 0.19 ^c^	7.43 ± 0.10 ^b^	8.11 ± 0.19 ^bc^
CN36	11.11 ± 0.05 ^abc^	11.37 ± 0.24 ^abc^	13.07 ± 0.21 ^de^	11.22 ± 0.03 ^abc^	11.08 ± 0.19 ^abc^	10.98 ± 0.81 ^ab^	10.69 ± 0.72 ^a^	11.06 ± 0.54 ^abc^	12.31 ± 1.78 ^cde^	14.88 ± 0.46 ^f^	12.01 ± 0.25 ^bcd^	13.36 ± 0.68 ^e^
CN38	12.85 ± 0.05 ^d^	13.07 ± 0.36 ^d^	10.14 ± 0.22 ^b^	13.16 ± 0.06 ^d^	12.91 ± 0.13 ^d^	9.68 ± 0.48 ^ab^	11.70 ± 1.11 ^c^	9.11 ± 0.52 ^a^	11.42 ± 0.82^c^	9.00 ± 0.63 ^a^	9.64 ± 0.33 ^ab^	10.08 ± 0.27 ^b^
CN40	10.10 ± 0.05 ^ab^	10.20 ± 0.10 ^ab^	11.02 ± 0.23 ^b^	10.24 ± 0.25 ^ab^	10.15 ± 0.13 ^ab^	19.09 ± 0.40 ^e^	10.62 ± 0.99 ^ab^	10.57 ± 0.24 ^ab^	12.69 ± 1.35 ^c^	15.68 ± 0.84 ^d^	9.48 ± 0.40 ^a^	12.34 ± 0.53 ^c^
CN42	7.38 ± 0.05 ^e^	7.18 ± 0.02 ^de^	7.09 ± 0.15 ^cde^	7.40 ± 0.09 ^e^	7.49 ± 0.08 ^e^	6.42 ± 0.44 ^b^	6.68 ± 0.08 ^bc^	6.72 ± 0.59 ^bc^	6.90 ± 0.22 ^cd^	5.61 ± 0.08 ^a^	6.35 ± 0.10 ^b^	5.73 ± 0.22 ^a^
CN44	7.03 ± 0.09 ^f^	6.78 ± 0.12 ^ef^	7.47 ± 0.27 ^g^	6.99 ± 0.10 ^f^	7.05 ± 0.09 ^f^	5.68 ± 0.24 ^a^	6.12 ± 0.38 ^bc^	6.97 ± 0.15 ^f^	6.38 ± 0.04 ^cd^	5.63 ± 0.06 ^a^	6.53 ± 0.15 ^de^	5.92 ± 0.14 ^ab^
CN46	7.48 ± 0.15 ^fgh^	7.29 ± 0.11 ^ef^	8.05 ± 0.11 ^i^	7.37 ± 0.09 ^fg^	7.43 ± 0.20 ^fg^	6.06 ± 0.22 ^a^	6.98 ± 0.37 ^de^	7.79 ± 0.17 ^hi^	6.69 ± 0.22 ^cd^	6.32 ± 0.06 ^ab^	7.67 ± 0.26 ^gh^	6.59 ± 0.09 ^bc^
CN48	9.35 ± 0.12 ^d^	8.98 ± 0.11 ^cd^	8.89 ± 0.18 ^cd^	9.20 ± 0.12 ^d^	9.23 ± 0.19 ^d^	7.52 ± 0.40 ^a^	8.97 ± 0.51 ^cd^	10.50 ± 0.31 ^e^	8.59 ± 0.55 ^bc^	7.64 ± 0.18 ^a^	10.35 ± 0.20 ^e^	8.29 ± 0.07 ^b^
CN50	10.92 ± 0.21 ^efg^	10.81± 0.30 ^defg^	9.48 ± 0.18 ^b^	10.76 ± 0.18 ^def^	10.90 ± 0.16 ^efg^	9.99 ± 0.28 ^bcd^	10.28 ±0.58 ^bcde^	11.65 ± 0.59 ^g^	10.38 ±1.04 ^cdef^	8.12 ± 0.60 ^a^	11.21 ± 0.10 ^fg^	9.71 ± 0.09 ^bc^
CN52	8.84 ± 0.08 ^def^	8.90 ± 0.20 ^def^	6.35 ± 0.32 ^a^	8.91 ± 0.20 ^def^	8.76 ± 0.10 ^def^	8.05 ± 1.15 ^cd^	9.42 ± 0.81 ^f^	8.38 ± 0.41 ^cde^	7.83 ± 0.23 ^bc^	7.15 ± 0.38 ^b^	9.13 ± 0.19 ^ef^	8.34 ± 0.11 ^cde^
CN54	3.76 ± 0.13 ^bcd^	4.03 ± 0.38 ^cd^	1.90 ± 0.23 ^a^	3.55 ± 0.32 ^bc^	3.67 ± 0.29 ^bc^	3.51 ± 0.66 ^bc^	4.49 ± 0.62 ^d^	3.49 ± 0.48 ^bc^	3.74 ± 0.74 ^bcd^	3.02 ± 0.16 ^b^	3.60 ± 0.27 ^bc^	3.48 ± 0.11 ^bc^

The data are expressed as means (g/100 g of fat ± standard deviation) of at least two replicates. Different superscripts in the same row indicate significant differences (*p* < 0.05). CN: carbon number. RWM: raw whole milk, PWM: pasteurized whole milk, PSKM: pasteurized skimmed milk, RC: raw cream, PC: pasteurized cream, BM1: buttermilk 1, BM2: buttermilk 2, BS1: butterserum 1, BS2: butterserum 2, BMP-1: buttermilk powder, BSP-1: butterserum powder, BM-BS BLEND: buttermilk and butterserum mix.

**Table 4:** Mean values of the lipid class profile of the selected samples and their corresponding MFGM isolates.

Lipid Classes	BM1	MFGM BM1	BM2	MFGM BM2	BS2	MFGM BS2	BMP-1	MFGM BMP-1	BM-BS BLEND	MFGM BM-BS BLEND
TAG	58.20 ± 0.80 ^a^	25.84 ± 1.50 ^b^	78.59 ± 6.68 ^a^	29.26 ± 0.86 ^b^	85.34 ± 0.26 ^a^	47.45 ± 0.73 ^b^	63.10 ± 0.93 ^a^	40.02 ± 0.87 ^b^	63.61 ± 3.02 ^a^	37.83 ± 2.27 ^b^
DAG	9.76 ± 0.56	12.38 ± 0.45	13.30 ± 5.63 ^a^	23.56 ± 0.96 ^b^	2.70 ± 0.28 ^a^	7.43 ± 0.92 ^b^	6.72 ± 0.49	8.51 ± 0.30	12.58 ± 1.59 ^a^	17.21 ± 2.09 ^b^
FFA CHOL	3.02 ± 0.24	3.38 ± 0.39	2.30 ± 0.97 ^a^	8.82 ± 0.67 ^b^	0.48 ± 0.02 ^a^	1.40 ± 0.21 ^b^	1.64 ± 0.06	1.58 ± 0.18	1.82 ± 0.14	2.43 ± 0.50
MAG	0.08 ± 0.01	0.07 ± 0.01	0.08 ± 0.03 ^a^	0.32 ± 0.09 ^b^	0.03 ± 0.00	0.07 ± 0.02	0.05 ± 0.02	0.06 ± 0.01	0.07 ± 0.01	0.09 ± 0.03
GLUCER	0.40 ± 0.08 ^a^	1.00 ± 0.03 ^b^	0.07 ± 0.01 ^a^	0.57 ± 0.01 ^b^	0.14 ± 0.02 ^a^	0.69 ± 0.07 ^b^	0.36 ± 0.02 ^a^	0.72 ± 0.10 ^b^	0.30 ± 0.07 ^a^	0.81 ± 0.05 ^b^
LACER	0.75 ± 0.05 ^a^	1.56 ± 0.06 ^b^	0.08 ± 0.00 ^a^	0.67 ± 0.14 ^b^	0.23 ± 0.00 ^a^	1.02 ± 0.12 ^b^	0.70 ± 0.04 ^a^	1.19 ± 0.13 ^b^	0.42 ± 0.15 ^a^	1.14 ± 0.05 ^b^
PL	27.79 ± 0.56 ^a^	55.69 ± 0.78 ^b^	5.57 ± 0.15 ^a^	36.69 ± 1.25 ^b^	11.08 ± 0.08 ^a^	41.60 ± 0.44 ^b^	27.42 ± 0.43 ^a^	47.66 ± 0.76 ^b^	19.33 ± 1.57 ^a^	40.35 ± 0.48 ^b^
NL	72.21 ± 0.56 ^a^	44.31 ± 0.78 ^b^	94.43 ± 0.15 ^a^	63.31 ± 1.25 ^b^	88.92 ± 0.08 ^a^	58.40 ± 0.44 ^b^	72.58 ± 0.43 ^a^	52.34 ± 0.76 ^b^	80.67 ± 1.57 ^a^	59.65 ± 0.48 ^b^

The data are expressed as means (g/100 g of fat ± standard deviation) of at least two replicates. Different superscripts in the same row indicate significant differences (*p* < 0.05). TAG: triacylglycerols; DAG: diacylglycerols; FFA + Chol: free fatty acids + cholesterol; MAG: monoacylglycerols; GlucCer: glucosylceramide; LacCer: lactosylceramide; PL: polar lipids; NL: neutral lipids.

**Table 5:** Identification of MFGM proteins via in-gel digestion of electrophoretic bands.

Band	Tryptic Peptide (Ion)	Protein Fragment	Sequence	Ion Score	Protein Name	Entry Number	Mascot Score
A	1216.84	949–958	K.EGDLTHFNQR.L	50	XDH	P80457	70
A	1334.96	1229–1240	K.IPAFGSIPTEFR.V	35	XDH	P80457	70
D	961.87	70–78	K.VSPAVFVSR.E	47	BTN	P18892	131
D	1018.94	411–420	R.TPLPLAGPPR.R	38	BTN	P18892	131
D	1077.85	368–376	R.TDWAIGVCR.E + Carbamidomethyl (C)	27	BTN	P18892	131
D	1124.97	332–340	R.QKLPEKPER.F	27	BTN	P18892	131
D	1254.8782	341–350	R.FDSWPCVMGR.E + Carbamidomethyl (C)	40	BTN	P18892	131
D	1255.8504	193–203	R.NPDEEGLFTVR.A	35	BTN	P18892	131
D	1276.9567	358–367	R.HYWEVEVGDR.T	46	BTN	P18892	131
E	1121.1414	409–417	R.IQPVAWHNR.I	35	PAS 6	Q95114	127
E	1194.1710	207–216	R.QFQFIQVAGR.S	61	PAS 6	Q95114	127
E	1223.1538	396–405	K.NIFETPFQAR.F	32	PAS 6	Q95114	127
E	1440.2549	349–360	R.DFGHIQYVAAYR.V	75	PAS 6	Q95114	127
F	842.7880	140–146	R.WAPELAR.L	27	PAS 7	Q95114	132
F	1007.9321	409–417	R.LVPIICHR.G + Carbamidomethyl (C)	16	PAS 7	Q95114	132
F	1120.9565	207–216	R.IQPVAWHNR.I	48	PAS 7	Q95114	132
F	1193.9977	396–405	R.QFQFIQVAGR.S	78	PAS 7	Q95114	132
F	1222.9794	175–187	K.NIFETPFQAR.F	34	PAS 7	Q95114	132

XDH: xanthine dehydrogenase/oxidase, BTN: butyrophilin subfamily 1 member A1, PAS 6 and PAS7: glycosylation variants of lactadherin.

## Data Availability

Not applicable.
